# Further strategies after immune checkpoint inhibitors in relapsed/refractory Hodgkin lymphoma: salvage treatments and consolidation with transplantation, experience in daily clinical practice

**DOI:** 10.1007/s00277-025-06255-8

**Published:** 2025-03-01

**Authors:** Cinzia Pellegrini, Beatrice Casadei, Alessandro Broccoli, Martina Cantelli, Gabriele Gugliotta, Marianna Gentilini, Matteo Carella, Vittorio Stefoni, Nicole Fabbri, Giulia Gabrielli, Lisa Argnani, Camilla Mazzoni, Pierluca Maglio, Gianmarco Bagnato, Pier Luigi Zinzani

**Affiliations:** 1 IRCCS Azienda Ospedaliero-Universitaria di Bologna, Istituto di Ematologia “Seràgnoli”, Via Massarenti, 9, Bologna, 40138 Italy; 2 Dipartimento di Scienze Mediche e Chirurgiche, Università di Bologna, Bologna, Italy

**Keywords:** Checkpoint inhibitors, Relapsed/refractory Hodgkin lymphoma, Salvage treatments, Consolidation, Real-world

## Abstract

Immune checkpoint inhibitors (CPIs) emerged as an effective and safe therapeutic option for patients with relapsed/refractory (R/R) classical Hodgkin lymphoma (cHL). However, many patients lose their response to CPIs. To improve outcomes in this setting, two strategies can be considered: salvage chemotherapy (CHT) for those with an unsatisfactory response, and autologous stem-cell transplantation (auto-SCT) as a consolidation for patients with at least partial response (PR).

We analyzed retrospectively the effectiveness in terms of rate of response and survivals of these two approaches in cohort 1 (salvage CHT) and in cohort 2 (auto-SCT consolidation). Adverse events were also assessed.

A total of 45 heavily pre-treated patients were analyzed (median of 4 prior therapies; 93.3% refractory to the last therapy). Thirty patients received further CHT at a median of 32 days (range 1-1213) after CPI Fifteen patients in cohort 2 underwent auto-SCT, with 8 in complete response (CR) and 7 in PR. In cohort 1 a final overall response rate (ORR) of 50.0% with a median progression-free survival of 24.6 months. In cohort 2, the ORR was 93.3% (all CR) with median survivals not reached. No unexpected or cumulative toxicities were observed.

Our findings suggest that auto-SCT is an effective consolidation strategy for cHL patients who achieve at least a PR after CPI therapy, despite multiple prior lines of treatment. Additionally, CPIs treatment appears to sensitize heavily pre-treated and chemorefractory patients to subsequent chemotherapy, potentially facilitating successful transplant consolidation and improving the chances of cure.

**Supplementary Information:**

The online version contains supplementary material available at 10.1007/s00277-025-06255-8.

## Introduction

Classical Hodgkin lymphoma (cHL) is generally considered a highly curable disease, however approximately 15–25% of patients do not respond to or relapse after conventional front-line treatment [1–4]. Traditionally, patients with relapsed/refractory (r/r) disease received non-cross-resistant high dose chemotherapy (HDT), including platinum-based regimens such as the ICE scheme (ifosfamide, carboplatin, and etoposide) [5], gemcitabine-based treatment such as the GVD scheme (gemcitabine, vinorelbine, and pegylated liposomal doxorubicin) [6], or less toxic regimen BEGEV (bendamustine, gemcitabine, vinorelbine) [7, 8] followed by consolidation with autologous stem cell transplantation (auto-SCT) in chemo-sensitive cases [9]. The combination of multi-agent chemotherapy (CHT) with auto-SCT resulted in a long-term cure in about 50% of patients [10]. On the contrary, the prognosis of patients who do not respond to second-line chemotherapy or who relapse after auto-SCT remains poor, with a median overall survival (OS) of only 2 years [10–12].

Several elements have been recognized as prognostic factors of auto-SCT outcome. Specifically, in the pre-PET (Positron Emission Tomography) era, primary refractory disease, early relapse within one year of first line treatment, and the presence of B symptoms or extra-nodal disease at relapse were associated with worse outcome [13]. Nowadays, however, PET status at the time of auto-SCT is considered the strongest predictor of patient prognosis [14].

Certainly, the approval over the last decade of novel drugs such as brentuximab-vedotin (BV), a drug-conjugated anti-CD30 monoclonal antibody [15] and nivolumab or pembrolizumab, both programmed death-1 (PD-1) inhibiting antibodies [16, 17], improved outcomes in r/r cHL patients [18–24]. However, these drugs showed limited complete response (CR) rates and did not provide sustained responses [19–24]. For instance, BV demonstrated an overall response rate (ORR) of 75%, with a CR in 33% (34/102) of patients with r/r HL after auto-SCT [19]. However, only 38% of CR patients (13/34) maintained a complete remission after 5 years of follow-up, with most patient requiring additional treatment within 1 year [20]. In the CheckMate-205 trial, nivolumab showed an ORR of 69% and a CR rate of 16% in 243 cHL patients after failure of auto-SCT or both auto-SCT and BV [21, 22]. Similarly, in the phase II KEYNOTE-087 study, pembrolizumab lead to achieve an ORR of 72% with a CR rate of 28% in 210 r/r cHL patients [23]. Moreover, the 5 year follow-up of these phase 2 trials suggests that about half of patients in CR are likely to eventually relapse, with a median progression free survival (PFS) for overall population of 15.1 and 13.7 months for nivolumab and pembrolizumab, respectively [21, 24]. In this context, identifying an appropriate strategy after PD-1 inhibition remains a challenge for both anti-PD1 responsive and refractory patients. Retrospective studies have shown a potential role of nivolumab and pembrolizumab in re-sensitizing tumor cells to CHT, leading to patients previously chemo-refractory and unresponsive to anti-PD1, to achieve a response after re-treatment with CHT used in combination or sequentially to check-point inhibitors (CPIs) [25–27]. Additionally, the efficacy of auto-SCT as a consolidation treatment, even in cases of partial remission (PR) following anti-PD1 exposure, has been supported by a large retrospective study and corroborated by our previously published experience [27, 28].

Based on previous findings and insights from our daily clinical practice, we explored two strategies to assess their potential in improving patient outcomes following CPI therapy: (1) the role of salvage CHT for patients who exhibit an unsatisfactory response to CPI treatment, and (2) the role of auto-SCT as a consolidation therapy for patients who achieve at least a PR with CPI.

## Methods

We retrospectively analyzed the outcome of salvage CHT (cohort 1, *N* = 30) rather than consolidation with auto-SCT (cohort 2, *N* = 15) in 45 patients with cHL who were treated with anti-PD1 therapy (pembrolizumab or nivolumab) from March 2015 to October 2022, achieving either an unsatisfactory response (PR, stable disease [SD] or progression disease [PD]; cohort 1) or a CR/PR deemed suitable for transplant consolidation (cohort 2). Regarding salvage approaches in cohort 1, the choice between multi-agent or single-agent CHT, or transplantation, was mainly made both on the basis of therapies performed prior to CPIs, avoiding re-treatment with agents already administered when possible, and on the basis of clinical characteristics such as age, performance status and tolerance to previous treatments. The patient list was extracted from the electronic database of our Institute. The study was approved by our institutional board and by our Ethical Committee and has been performed in accordance with the ethical standards as laid down in the 1964 Declaration of Helsinki and its later amendments (Ethical Committee AVEC of Bologna, approval id 1043/2021/Oss/AOUBo). Patients were consecutively enrolled to avoid selection bias, and all patients provided written informed consent to collect retrospectively their data when applicable. We obtained a special permission (for scientific purpose) from our Ethical Committee to collect even data of patients who were deceased or lost to follow-up. To be enrolled patients must have received at least two cycles of single agent nivolumab or pembrolizumab and must have unsatisfactory response (cohort 1) or achieved at least a PR (cohort 2) to CPI. The diagnosis of cHL was established from lymph node biopsies, in accordance with the 2008 World Health Organization classification [29]. In both cohorts, responses were assessed with PET scan and computed tomography (CT) scan, with different timings depending on the type of treatment the patient was undergoing. ORR (defined as the sum of CR and PR at the end of treatment) and complete response rate (CRR) in both cohorts were chosen as primary endpoint, whereas PFS and OS were analyzed as secondary endpoints. OS was defined as the time from first cycle of therapy (CHT) performed after CPI to death from any cause and was censored at the date of last available follow up. PFS was measured from initiation of therapy performed after CPI to progression, relapse, or death from any cause [30]. Responses were classified according to the Lugano criteria [31, 32]. The toxicities were graded according to the National Cancer Institute Common Toxicity Criteria for Adverse Events (CTCAE version 4.0). No formal sample size estimation and power calculation were made for this observational retrospective study as we enrolled all patients treated at our Institute according to study inclusion criteria. Demographics and patients’ characteristics were summarized by descriptive statistics. Continuous and categorical variables were presented as median (range) and n (%), respectively. Comparisons were executed using the Student’s t or Mann-Whitney tests for continuous variables and a Chi-square or Fisher’s exact test for categorical variables, as applicable. Survival functions were estimated by using the Kaplan-Meier method and were compared using log-rank test. Confidence intervals (CI) at 95% were provided. Statistical analyses were performed in Python version 3.10.12 (including the following packages: lifelines version 0.29.0, numpy version 1.26.4, pandas version 2.1.4, scipy version 1.13.1) and *p* values for statistical significance were set at 0.05. Matplotlib version 3.7.1 was used to create charts.

## Results

### Patients

Thirty patients in cohort 1 and 15 patients in cohort 2 were enrolled. No statistically significant differences in patient characteristics were observed between the two cohorts, with the exception of a higher number of patients refractory to first-line therapy in cohort 1 (83.3% vs. 66.7%, respectively; *p* < 0.05) (Table 1). Considering the total of 45 patients enrolled, 53.3% (*N* = 25) were females, with a median age at diagnosis of 29.9 years (range 17.6–69.8). All patients but one received first-line therapy according to ABVD (doxorubicin, bleomycin, vinblastine, dacarbazine), to which 77.8% (*N* = 35) were refractory. The study population was highly pretreated with a median of four therapies (range 1–9) before CPI, including auto-SCT (22%), radiotherapy (22%) and BV (89%) (Table 1). There was a high heterogeneity in the treatment given immediately before CPI, with the most common being BV as single agent (25 patients, 55%) or in combination with chemotherapy (3 patients, 6.6%). At the start of anti-PD1 therapy, the median age was 32 years (range 19.3–72), with the majority of patients (93.3%, *N* = 42) being refractory to the last therapy before CPI. Thirty out of 45 patients received pembrolizumab (3 patients at the dose of 10 mg/kg and 27 at the flat dose of 200 mg every 3 weeks) and the remaining underwent nivolumab (9 patients at the dose of 3 mg/kg and 6 at the flat dose of 200 mg every 2 weeks). A median of 14 cycles (range 3–52) of anti-PD1 therapy were infused (Table 1). The best ORR obtained with CPI was 71,1%, with 8 patients achieving a CR and 24 a PR. At the end of treatment, 62.2% of patients did not respond to anti-PD1 therapy (25 PD and 3 SD), whereas ten had a PR and seven patients achieved a CR, with an ORR of 37.8% (Table 1).

**Table 1:** Patient characteristics

Patient characteristics	Total population (*n* = 45)	Cohort 1 Salvage chemotherapy (*n* = 30)	Cohort 2 ASCT (*n* = 15)	*P*
Female, n (%)	24 (53.3)	17 (56.7)	7 (46.7)	ns
Median age at diagnosis, years (range)	29.9 (17.6–69.8)	31.1 (17.6–69.8)	28.9 (19.8–41.3)	ns
Histologic subtypes, n (%)				ns
Nodular sclerosis	42 (93.3)	27 (90)	15 (100)	
Mixed cellularity	2 (4.4)	2 (6.7)	0	
Lymphocyte-depleted	1 (2.2)	1 (3.3)	0	
Ann Arbor stage at diagnosis, n (%)				ns
I-II	22 (48.9)	13 (43.3)	9 (60)	
III-IV	21 (46.7)	17 (56.7)	6 (40)	
Unknown	2 (4.4)	0	0	
Prior therapies to CPi, median (range)	4 (1–9)	4 (1–9)	3 (1–5)	ns
ASCT, n (%)	10 (22.2)	10 (33.3)	0	NA
Radiotherapy, n (%)	10 (22.2)	7 (23.3)	3 (20)	ns
Brentuximab Vedotin, n (%)	40 (89)	27 (90)	13 (87)	ns
Outcome of first line therapy, n (%)				
Refractory	35 (77.8)	25(83.3)	10 (66.7)	< 0.05
Relapsed	10 (22.2)	5 (16.7)	5 (33.3)	ns
Median age at CPIs, years (range)	32.0 (19.3–72.0)	34.2 (19.3–72.0)	31.6 (20.8–44.2)	ns
Ann Arbor stage at CPIs, n (%)				ns
I-II	21 (46.7)	13 (43.3)	8 (54.4)	
III-IV	24 (53.3)	17 (56.7)	7 (46.6)	
Outcome of last therapy before CPIs, n (%)				
Refractory	42 (93.3)	28 (93.3)	14 (93.3)	ns
Relapsed	3 (6.7)	2 (6.7)	1 (6.7)	
Type of CPIs received, n (%)				ns
Nivolumab	15 (33.3)	10 (33.3)	5 (33.3)	
Pembrolizumab	30 (66.7)	20 (66.7)	10 (66.7)	
Number of cycle of CPIs, median (range)	​14 (3–52)	14 (3–52)	​12 (6–49)	< 0.05
Best ORR to CPIs, n (%)	32 (71.1)	17 (56.6)	15 (100)	0.0018
Final ORR to CPIs, n (%)	17 (37.8)	2 (6.7)	15 (100)	< 0.0001

ASCT: autologous stem cell transplantation; CPi: check point inhibitors; ORR: overall response rate

### Outcome and toxicity of treatment after CPIs

**Fig. 1:**
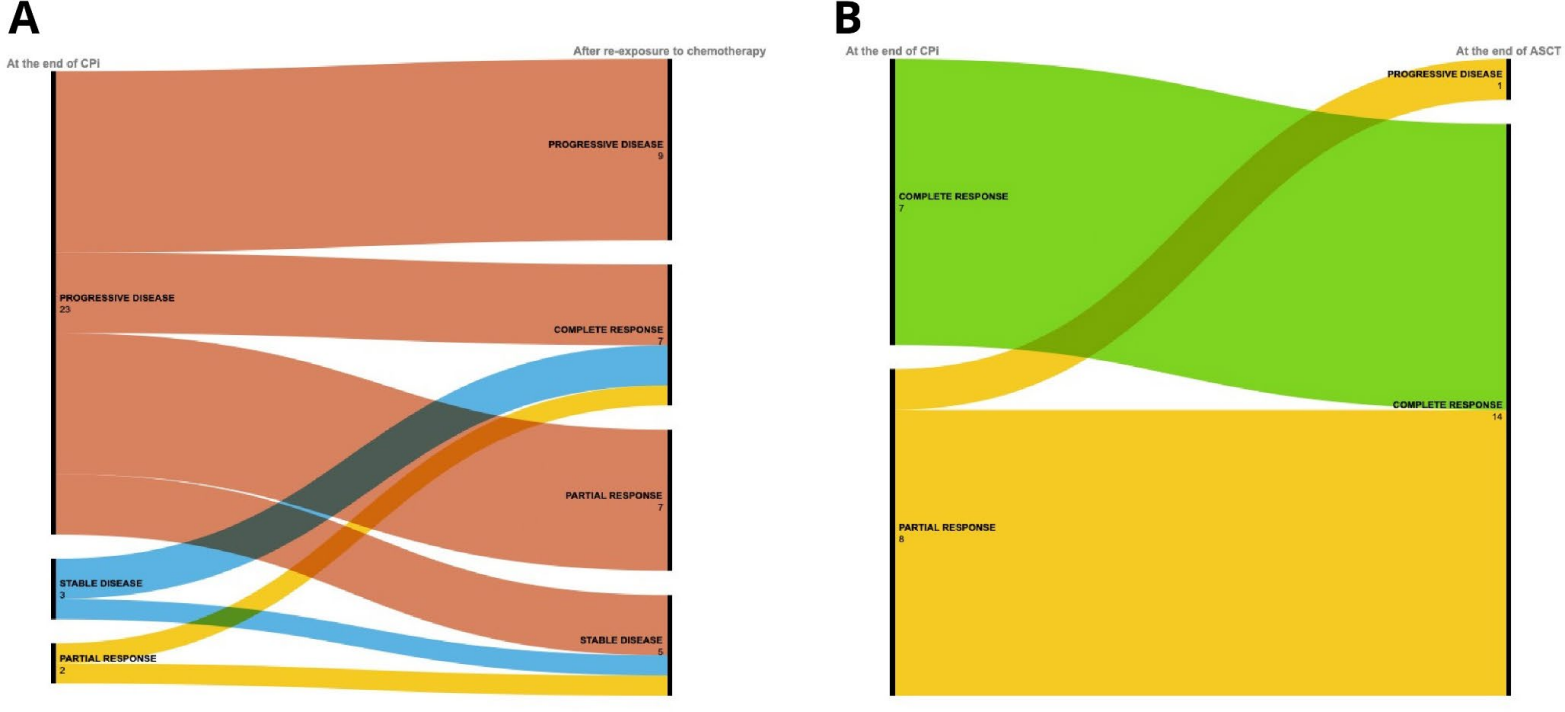
Conversion of response in cohort 1 and 2. **A** Conversion of response after re-exposure to chemotherapy in patients with unsatisfactory response to CPi, focus on cohort 1; **B** Conversion of responses after autologous stem cell transplantation, focus on cohort 2

**Fig. 2:**
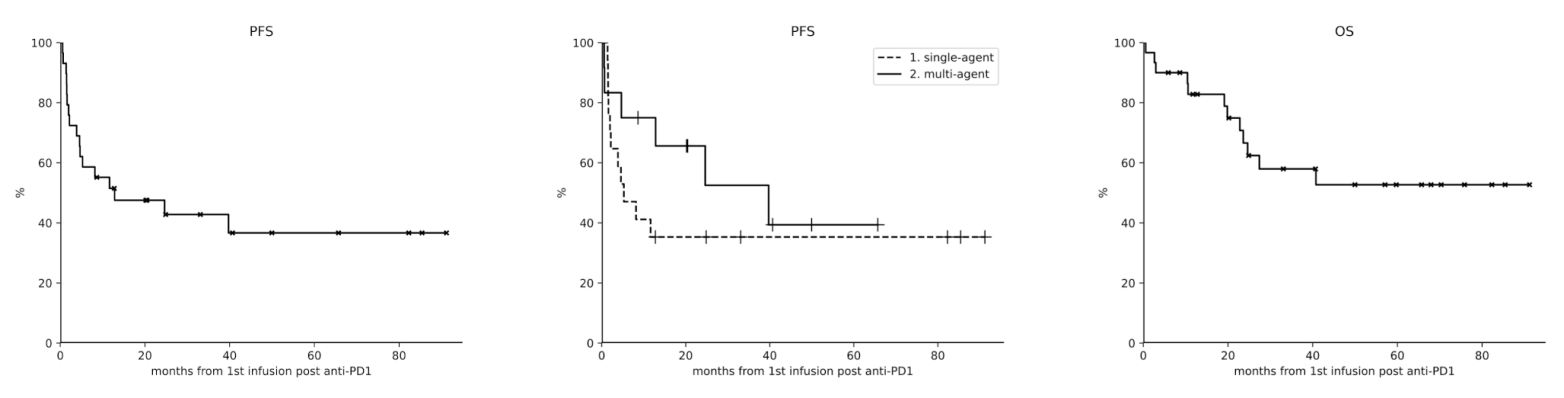
Outcome of patients in cohort 1. **A** Progression free survival. **B** Progression-free survival with salvage treatment (1: single agent; 2: multi-agents regimen). **C** Overall survival

After a median time of 32 days (range 1-1213) from the last response assessment to anti-PD1 treatment, 17 out of 30 patients (56.7%) in cohort 1 received a single agent CHT, whereas 12 (40%) had a multi-agent treatment (Table S1). Twenty-eight out of thirty patients were evaluable for response, thus, after a median of three cycles (range 1–10), 7 patients obtained a CR (CRR: 25%) and 7 a PR, with an ORR of 50% (Table 2; Fig. 1A). Patients undergoing multi-agent treatment had a significant higher ORR than those treated with single agent therapy (ORR: 81.82% vs. 29.41%, *p* = 0.0202). Furthermore, seven patients were re-exposed to the same CHT agents that they have received before check-point inhibition, all of them were refractory at the first exposure and five out of seven (71.4%) became responsive after anti-PD1 treatment (Table S1). Twenty-three patients (82.1%) discontinued the salvage treatment: eleven due to unsatisfactory response at the first evaluation, five due to a grade 2–4 toxicity and seven patients due to a consolidation with stem cell transplantation. Specifically, 3 patients (one in CR and two in PR) received auto-SCT while the remaining four underwent to allogenic stem cell transplantation (allo-SCT, three patients were in CR and one in PR). Transplantation as a consolidation strategy allowed all PR patients to convert their response into CR. Overall, 13 (46.4%) of the 28 patients who failed treatment with CPIs achieved a CR, with a median of two subsequent lines of salvage CHT (range 1–4) (Table S1). In term of toxicity, 11 (50%) experienced hematological side effects: seven patients had grade 3–4 neutropenia, four patients had thrombocytopenia (1 grade 2 and 3 grade 3–4, respectively) while two patient had grade *≤* 2 anemia. Seven patients (31.9%) presented at least one extra-hematological AE, predominantly grade 1–2 (82.3% of events). Specifically, the most frequent grade *≤* 2 event was a skin rash occurring in 5 out of 7 patients. One patient died before response evaluation due to febrile neutropenia and pneumonia of grade 5. Median PFS with salvage treatment was reached at 12.8 months (95% CI, 3.3 to not reached) (Fig. 2A) with no statistically differences between multi-agent and single agent regimen: 39.7 months (CI 95%, 0.6 to not reached) vs. 5.2 months (CI 95%, 1.6 to not reached), *p* = 0.32 (Fig. 2B). PFS estimated from start of salvage therapy was 42.8% at 3 years (95% CI: 24.1–60.3%). After a median follow-up of 24.8 months, 12 patients died (eight due to a PD, one due to febrile neutropenia and pneumonia, one in CR due to secondary acute myeloid leukemia and the last one also in CR due to pneumonia) while 11 patients are still alive and in CR, with an estimated OS at 3 years of 58% (median not reached, 95% CI: 36.7–74.4%) (Fig. 2C).

**Table 2:** Response rates to treatment performed immediately after check-point inhibitors

Final response to first treatment after CPi	Total population (*n* = 43*)	Cohort 1 Salvage chemotherapy (*n* = 30)	Cohort 2 ASCT (*n* = 15)	*P*
ORR, n (%) CR, n (%) PR, n (%)	28 (65.1) 21 (48.8) 7 (16.3)	14 (50.0) 7 (25.0) 7 (25.0)	14 (93.3) 14 (93.3) 0 (0.0)	< 0.5
SD, n (%)	5 (11.6)	5 (17.9)	0 (0.0)	
PD, n (%)	10 (23.3)	9 (32.1)	1 (6.7)	

ASCT: autologous stem cell transplantation; CR: complete response; ORR: overall response rate; PD: progression disease; PR: partial response; SD: stable disease

* Two patients had non-assessable response, one due to an adverse event of grade 5 and one was lost at follow-up

**Fig. 3:**
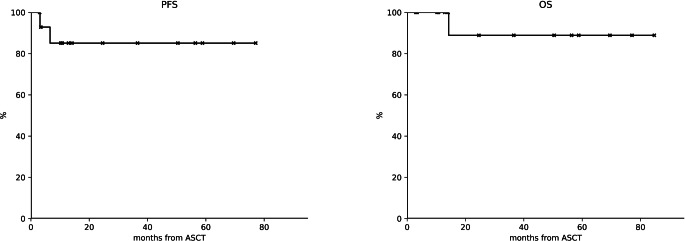
Outcome of patients in cohort 2. **A** Progression free survival. **B** Overall survival

Regarding the 15 patients who underwent to autologous transplantation as consolidation treatment after PD-1 blockade (cohort 2), 14 achieved a CR (CRR: 93.3%), specifically 7 converted their PR obtained with CPI into CR, with an ORR to auto-SCT of 93.3% (Fig. 1B). Thirteen patients were evaluable for toxicity, all of whom experienced at least one hematological AE of any grade. Specifically, 13 patients had grade 4 neutropenia, of which 7 had febrile neutropenia, while 12 had grade 4 thrombocytopenia, requiring transfusion support. All hematological events resolved with a median of 5 and 4 days, respectively. Fifteen non-hematological AE were observed, all except one of grade 2 or less. The most common were mucositis in eight patients (seven of grade 2 and one of grade 3) and diarrhea in two patients. None of them developed engraftment syndrome. In the four patients who subsequently underwent allo-SCT after at least one line following CPi there were no cases of severe GVHD. With a median follow-up of 24.6 months, both median PFS and OS were not reached. In particular, PFS estimated at 3 years from auto-SCT was 85.1% (CI 95%: 52.3–96.1%) (Fig. 3A) while the OS was 88.9% (CI 95%: 43.3–98.4%), respectively (Fig. 3B). Comparing the PFS between the two populations, those in CR and those in PR prior to ASCT, no differences were observed.

## Discussion

CPIs have significantly enriched the therapeutic options for patients with r/r cHL. This improvement is not only reflected in the proportion of patients achieving a response thanks to CPIs, but also in the peculiar chemosensitization effect observed in previously refractory patients.

The mechanisms driving this chemosensitizing effect are not yet fully understood. One theory proposes that CPIs may affect the tumor microenvironment (TME) in a way that enhances chemotherapy effectiveness, possibly by disrupting pro-survival signals within the TME [33]. Chemotherapy may augment cytotoxic T-lymphocytes activity by increasing tumor antigen presentation and facilitating the infiltration of these lymphocytes into the tumor tissue [34]. Additionally, studies in mice suggest that chemotherapy can elevate the mutational load, thereby enhancing the effectiveness of checkpoint blockade therapy [35]. Compelling evidence in cHL confirms that CPIs enhance chemo-responsiveness in previously chemo-refractory patients. Rossi and colleagues reported an ORR of 66% and a median PFS of 11 months in 30 chemo-refractory patients after re-treatment with CHT, in association with or immediately after check-point inhibition [26]. Notably, 15 patients were re-exposed to the same chemotherapy regimen, including 6 who had been initially refractory. Similar results were observed in our previous retrospective study [27], where we assessed salvage chemotherapy in 25 r/r cHL patients with unsatisfactory responses to anti-PD1 therapy. Treated with either multi-agent or single-agent regimens, the ORR was obtained in 60% of patients, with 8 reaching a CR. Positive results were also observed when auto-SCT is used as a consolidation strategy in patients responsive to anti PD-1 therapy but previously chemo-refractory, suggesting that the demonstration of chemosensitivity prior to auto-SCT may not be essential. In our first retrospective case series, we showed that consolidation with auto-SCT after CPI resulted in CRR of 84.6%, with a 5-year disease-free survival of 87.5% [36]. Similarly, Merryman et al. retrospectively reviewed 78 high-risk, multiply r/r cHL patients who underwent auto-SCT following CPI therapy. Despite a high prevalence of chemorefractory disease in this cohort, including 41% of patients with positive pre-transplantation PET-CT scans, the outcomes were notably favorable, with an 18-month PFS rate of 75% [28]. Based on the high overall efficacy and potential chemosensitizing effect of CPIs, recent studies have explored the association between anti PD-1 treatment and second line HDT, showing high rates of complete metabolic remission. For example, the combination of pembrolizumab with GVD achieved a 100% ORR and a 95% of CRR, with 92% of patients in CR after two cycles and 95% proceeding directly to auto-SCT [37]. Similarly, the combination of nivolumab with ICE (NICE) in a phase II trial resulted in over 90% of CR, enabling patients to proceed to auto-SCT [38]. A “chemotherapy-free regimen” of BV with nivolumab (nivo-BV) was tested in a phase I/II study with 61 r/r cHL patients. After four cycles of nivo-BV, 92% of patients were able to proceed to auto-SCT, with 74% doing so directly. With a median follow-up of 34.3 months, the estimated 3-year PFS was 77% overall, while 91% in those who underwent directly to auto-SCT [39].

In Italy, none of these novel combination therapies is currently approved, making the treatment of patients with r/r cHL a significant challenge in clinical practice. In our study we analyze a real-life retrospective clinical experience focus on the chemosensitizing ability of the anti-PD1 drug class. Within the study population we identified two groups with dinstict characteristics. Cohort 1 included patients who exhibited an unsatisfactory response (PR, SD) or progression disease with CPIs, with primary focus on chemo-salvage therapies post-CPIs and their subsequent outcomes. Cohort 2 included patients who achieved a CR or PR after treatment with CPIs deemed satisfactory to proceed with ASCT, emphasizing the role of ASCT in patients who responded to CPIs. Cohort 1 results indicated that 50% of patients who received conventional chemotherapy after failing CPI therapy achieved an objective response (Table 2; Fig. 1A). Remarkably, seven patients were re-treated with the same chemotherapy agents they had received before checkpoint inhibition. Despite being refractory during the initial treatment, five of these seven patients (71.4%) responded after anti-PD1 therapy (Table S1). Additionally, using transplantation as a consolidation strategy allowed all patients who achieved a PR after salvage CHT to convert their response into a CR. Among the 28 patients who did not respond to CPIs, 46.4% reached a CR after a median of two additional lines of salvage chemotherapy (range 1–4) (Table S1). These findings, in line with previous studies, confirm that while some patients may not respond to anti-PD1 therapy initially, it can potentially restore chemosensitivity. This restoration offers the possibility of curing r/r disease through a bridge to autologous-SCT or, in some cases, allo-SCT. Cohort 2 results further support the use of transplantation as a consolidation strategy after anti PD-1, converting PR to CR and yielding positive outcomes even in patients with high-risk factors prior to auto-SCT. Regarding allo-SCT, in our cohort, there were no cases of severe GVHD This outcome was expected, considering that anti-PD1 therapy was not the last line of treatment before transplantation in this four patients. Additionally, the response to a salvage chemotherapy regimen (cohort 2) in relapsed/refractory patients with high-risk characteristics, including post-autologous relapse, can be explained by the chemosensitizing ability of anti-PD1 therapy.

Our study, while limited by its retrospective design and small sample size, offers valuable insights into the treatment of r/r cHL, a condition that presents significant clinical challenges. Our real-world evidence underscores the critical role of CPIs, which, despite their limited CR rate, appear to enhance chemosensitization for subsequent chemotherapy, leading to important practical implications. Firstly, auto-SCT emerges as an effective consolidation strategy for patients with cHL who achieve at least a PR after CPI therapy. Even in cases with multiple prior treatment lines and chemorefractoriness, auto-SCT should be prioritized over allo-SCT, especially in those patients who previously didn’t undergo to auto-SCT. Secondly, it is noteworthy that heavily pre-treated, chemorefractory r/r cHL patients who progress during CPIs treatment may still respond to subsequent therapies.

While anti PD-1 therapy is going to be increasingly integrated into frontline and rescue treatment regimens, it remains crucial in the current setting to maintain awareness of chemosensitization. This underscores the importance, in the post-CPI treatment context, of salvage chemotherapy on one hand and of auto-SCT as a consolidation strategy on the other hand. Recent studies suggest that immunotherapy is likely to become a standard component of first- or second-line treatment, and we expect that this will lead to improved patient outcomes. A novel question that will emerge is determining which patients genuinely require consolidation with transplantation, as more effective combination therapies become available in the rescue setting.

## Electronic supplementary material

Below is the link to the electronic supplementary material.

Supplementary Material 1

## Data Availability

The datasets used and/or analyzed during the current study are available from the corresponding author on reasonable request.
