# Supplementary material for: Further strategies after immune checkpoint inhibitors in relapsed/refractory Hodgkin lymphoma: salvage treatments and consolidation with transplantation, experience in daily clinical practice
Source: Ann Hematol. 2025 Mar 1;104(3):1757–64. doi: 10.1007/s00277-025-06255-8 (PMC12031856; doi:10.1007/s00277-025-06255-8)
Supplement: Supplementary file 1 — Supplementary Material 1 [file 277_2025_6255_MOESM1_ESM.docx]

**Table S1.** Treatment after check-point inhibitor failure

| Pts | Treatment before CPi | Response | CPi, type | Response | Age at I line of therapy post CPi | I line of therapy post CPi | N of cycles | Response | Re-exposure to the same treatment | SCT | Response to SCT | Last follow-up |
| --- | --- | --- | --- | --- | --- | --- | --- | --- | --- | --- | --- | --- |
| 1 | BV | PD | Pembrolizumab | PD | 56 | BeGeV | 4 | CR | Bendamustine, gemcitabine |  |  | CR |
| 2 | Bendamustine-BV | PD | Pembrolizumab | PD | 60 | BEACOPP | 3 | PR | Doxorubicin | auto-SCT | CR | CR |
| 3 | BV | PD | Pembrolizumab | PD | 28 | Bendamustine | 3 | PD | no |  |  | SD |
| 4 | BV | PD | Nivolumab | PD | 29 | Bendamustine | 6 | PD | no |  |  | CR |
| 5 | Bendamustine-BV | PD | Nivolumab | PD | 32 | Doxorubicin hydrocloride | 1 | SD | no |  |  | PD |
| 6 | BV | PD | Nivolumab | PD | 26 | DHAP | 3 | PR | Cytarabine | allo-SCT | CR | CR |
| 7 | BEAM | SD | Nivolumab | PD | 25 | Liposomal daunorubicin | 2 | PR | no |  |  | CR |
| 8 | Clinical trial | PD | Pembrolizumab | PD | 45 | Doxorubicin hydrocloride | 3 | SD | no |  |  | PD |
| 9 | Bendamustine-BV | PD | Pembrolizumab | PD | 34 | Doxorubicin hydrocloride | 5 | PR | no | auto-SCT | CR | CR |
| 10 | DHAP | PD | Pembrolizumab | SD | 74 | EDO101 | 10 | SD | no |  |  | PD |
| 11 | BV | PD | Pembrolizumab | PD | 41 | EDO101 | 6 | PD | no |  |  | ND |
| 12 | Gemcitabine | PD | Pembrolizumab | PR | 42 | EDO101 | 6 | SD | no |  |  | PD |
| 13 | Bendamustine | CR | Pembrolizumab | PD | 31 | Doxorubicin hydrocloride | 4 | CR | no | allo-SCT | CR | CR |
| 14 | Vinblastine | PD | Nivolumab | PD | 49 | Doxorubicin hydrocloride | 2 | PD | no |  |  | PD |
| 15 | DHAP | PD | Pembrolizumab | PD | 35 | ICE | 2 | PR | no |  |  | PD |
| 16 | BeEAM | PD | Nivolumab | SD | 31 | IGeV | 3 | CR | no | allo-SCT | CR | CR |
| 17 | BV | PD | Pembrolizumab | PD | 38 | BEAM | 1 | CR | Etoposide |  |  | CR |
| 18 | FEAM | PD | Nivolumab | PD | 57 | BeGeV | 3 | CR | no | allo-SCT | CR | CR |
| 19 | ABVD | PD | Pembrolizumab | PR | 20 | BeGeV | 1 | CR | no | auto-SCT | CR | CR |
| 20 | BV | PD | Pembrolizumab | PD | 71 | IEV | 1 | PD | Vinblastine |  |  | PD |
| 21 | BV | PD | Nivolumab | PD | 22 | Doxorubicin hydrocloride | 6 | PD | no |  |  | PD |
| 22 | BV | PD | Pembrolizumab | PD | 31 | Doxorubicin hydrocloride | 3 | PD | no |  |  | PD |
| 23 | BV | PD | Pembrolizumab | PD | 28 | Bendamustine | 1 | PD | no |  |  | PD |
| 24 | BV | PD | Pembrolizumab | PD | 57 | Camidanlumab | 5 | PR | no |  |  | CR |
| 25 | BV | PD | Pembrolizumab | PD | 19 | Doxorubicin hydrocloride | 3 | SD | no |  |  | PR |
| 26 | BV | PD | Nivolumab | PD | 25 | Camidanlumab | 3 | PR | no |  |  | CR |
| 27 | Bendamustine | CR | Pembrolizumab | SD | 26 | BeGeV | 2 | CR | Bendamustine |  |  | SD |
| 28 | BEAM | PD | Nivolumab | PD | 41 | BeGeV | 2 | PD | Gemcitabine |  |  | PD |
| 29* | BV | PD | Pembrolizumab | PD | 45 | Radiotherapy | unk | unk | no |  |  | unk |
| 30* | Bendamustine | PD | Pembrolizumab | PD | 55 | BEAM | 1 | unk | Etoposide |  |  | unk |

ABVD: doxorubicin, bleomycin, vinblastine, dacarbazine; allo-SCT: allogenic-stem cell transplantation; ASCT: autologous stem cell transplantation; BEACOPP: bleomycin, etoposide, doxorubicin, cyclophosphamide, vincristine, procarbazine, prednisone; BEAM: carmustine, etoposide, cytarabine, melphalan; BeEAM: bendamustine, etoposide, cytarabine, melphalan; BeGeV: bendamustine, gemcitabine, vinorelbine; BV: brentuximab vedotin; CPi: check point inhibitors; CR: complete response; DHAP: desametasone, high dose cytarabine, cisplatin; FEAM: fotomustine, etoposide, cytarabine, melphalan; ICE: ifosfamide, carboplatin, etoposide; IEV: ifosfamide, epirubicin, etoposide; IGeV: ifosfamide, gemcitabine, vinorelbine; ORR: overall response rate; N: number; PD: progression disease; PR: partial response; Pts: patients; SCT: stem cell transplantation; SD: stable disease.

* Patients with non-assessable response.
